# Effectiveness of Blog Writing Intervention for Promoting Subjective Well-Being, Resilience, and Post-Traumatic Growth of Palliative Care Nurses

**DOI:** 10.3390/healthcare13212757

**Published:** 2025-10-30

**Authors:** Nasreen Lalani, Gulnar Ali, Kawther Hamash, Aracely Ines Jimenez Paladines

**Affiliations:** 1 School of Nursing, Purdue University, West Lafayette, IN 47907, USA; 2 Department of Social Work Counselling & Social Care, School of Childhood and Social Care, University of East London, London E16 2RD, UK; g.ali@uel.ac.uk (G.A.); u2088519@uel.ac.uk (A.I.J.P.); 3 Wellstar School of Nursing, Kennesaw State University, Kennesaw, GA 30144, USA; khamash@kennesaw.edu

**Keywords:** nurse burnout, blog writing, self-reflexive practice, palliative care, resilience, personal growth, well-being

## Abstract

**Background/Objectives**: Palliative care nurses are at risk of burnout, trauma, and poor well-being. Expressive writing interventions are shown to promote self-reflection, personal growth, and resilience. A pilot study was designed to test the feasibility and use of a self-reflexive blog writing intervention to promote the subjective well-being, resilience, and personal growth of palliative care nurses during the COVID-19 pandemic. **Methods**: A sample of N = 144 registered nurses working in palliative care settings were enrolled in the study. Recruitment was performed using university alumni, nursing, and palliative care organization member listservs. Self-reported surveys such as the Subjective Well-Being Inventory (SUBI), Brief Resiliency Scale (BRS), and Post-traumatic Growth Inventory (PTGI) scales were used to evaluate study outcomes. Pre- and post-surveys were obtained at baseline, 4 weeks, and 6 weeks. Upon baseline survey completion, participants were randomly assigned to control and intervention groups. Participants in the intervention group were asked to write two weekly blog entries for a period of four weeks using a blog template on Qualtrics software. Descriptive statistical measures were used to evaluate the study outcomes and content analysis to analyze descriptive survey responses and blog narratives. **Results**: A total of N = 57 participants completed this study. Most participants were females (93%), White (89.5%), married (93%), and full-time employees (96.5%) and underwent PC training (93%). The findings indicate significant improvement in the subjective well-being (MD = 2.43, *p* < 0.05) and resilience scores (MD = 0.244, *p <* 0.05) in the intervention group. No significant changes were found in post-traumatic growth scores post-intervention. Most participants found blog writing as a supportive tool to reflect on their personal experiences and to ventilate their emotions and feelings. **Conclusions**: Self-reflexive blog writing is convenient and a potentially effective method for promoting the resilience and well-being of nurses. Future studies are needed to evaluate its effectiveness in a larger sample across different practice settings.

## 1. Introduction

Palliative care (PC) nurses find their work stressful and emotionally challenging and are at increased risk for burnout, trauma, and poor well-being [1]. PC nurses frequently experience loss, grief, and suffering providing care for dying patients and their families [2,3] and require continuous support for recovery and personal growth [1,3,4]. During the COVID-19 pandemic, nurses suffered increased post-traumatic stress, fear, anxiety, and depression across the globe [5,6,7]. About 40% of all COVID-19 deaths occurred in PC settings, such as nursing homes and long-term care, which include both healthcare staff and residents [8]. Reports indicate that more than 34% of healthcare workers including nurses are suffering from mild to severe post-traumatic stress disorder (PTSD) symptoms [9]. Nurses report guilt, self-doubt, anxiety, and depression and are finding it extremely hard to perform their clinical duties [7,10]. Not addressing these concerns may cause dysfunctional coping, suicide, substance use, and other harmful health-seeking behaviors among nurses [10,11,12,13,14]. Many healthcare agencies are witnessing high attrition rates, nursing shortages, and poor healthcare outcomes [2]. Effective strategies are needed to address the holistic well-being and overall quality of life of PC nurses [15].

Self-reflexive or expressive writing interventions such as those incorporating the use of journaling, blogs, poems, or art are found to promote self-reflection, creativity, and personal growth [16,17,18,19]. These activities allow individuals to express and process their emotions, feelings, and experiences to facilitate meaning making, resilience, and well-being [17,20]. Studies report that writing about personal experiences contributes positively to physical and mental health and promotes healing [17,18,21]. Self-reflexive writing interventions are flexible, non-invasive, and less threatening to use among different population groups [22]. However, these interventions are limited and require further investigation, particularly in a palliative care nursing and pandemic context. A pilot study was designed to assess the feasibility of a self-reflexive blog writing intervention to improve the subjective well-being, resilience, and post-traumatic growth outcomes of PC nurses during the COVID-19 pandemic.

### 1.1. Operational Definitions of Study Terms

Subjective well-being is defined as individuals’ self-reported perceptions of their overall happiness, life satisfaction, and emotional balance and is measured using the Subjective Well-Being Inventory (SUBI), which assesses both positive and negative affect, as well as satisfaction with various life domains. Resilience is the ability to bounce back from stress, as measured by the Brief Resilience Scale (BRS), which assesses individuals’ capacity to recover from adversity [23]. Personal growth is defined in terms of positive psychological change following adversity and is measured by the Post-traumatic Growth Inventory (PTGI), which captures growth in areas such as the appreciation of life, relationships, and personal strength [24].

### 1.2. Conceptual Framework

This study was guided by two theoretical frameworks: the Nurses Psychological Trauma (NPT) framework by Foli (2022) [11] and the Self-exploration through Ontological, Phenomenological, Humanistic, Ideological, and Existential expressions (SOPHIE) framework by Ali (2018) [25]. The NPT framework guided our understanding of nurse-specific traumas, the underlying factors, and how it influences health and well-being. According to NPT, nurses are highly vulnerable to nurse-specific trauma, as their roles are unique and differentiated from other healthcare providers [11]. These traumas can be classified as nurse-focused (personal) or patient-focused (professional), or these may overlap with each other, causing moral injury or psychological or existential crises. If these concerns are not addressed, this may cause nurses to engage in harmful health behaviors or attempt suicide, or it may predispose them to chronic mental health conditions.

**Figure 1:**
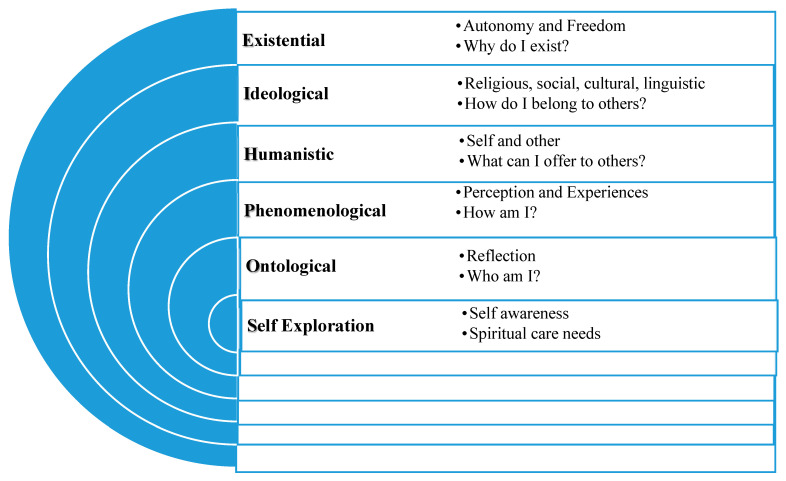
A framework for approaching spiritual care need.

Another framework, SOPHIE, discusses the principles of healing and empowerment through the existential and narrative inquiry process [25]. SOPHIE refers to self-exploration through one’s experiences, environment, values, and beliefs. It recognizes the unexplored behavioral patterns, belief systems, and intentionality that often represent one’s position in society and the nature of their relationships with others [26,27]. Allowing individuals to shape their own ontological spaces, and take responsibility for their own self-development, generates self-awareness, personal growth, and transformative healing [26]. (See Figure 1). These frameworks guided the development and evaluation of the intervention in this study.

## 2. Methods

### 2.1. Study Design

A pilot mixed-method study was designed to evaluate the feasibility, acceptability, and preliminary efficacy of a self-reflexive blog writing intervention to improve the subjective well-being, resilience, and personal growth of PC nurses.

### 2.2. Sample and Recruitment

For this study, a convenience sample of registered nurses working in PC settings (i.e., nursing homes, hospices, and long-term care settings) was recruited. This study was conducted during the pandemic between October 2021 and April 2023. Participant recruitment was performed using a university alumni listserv, social media posts, websites, and member listservs of nursing, palliative, and hospice care organizations. The PI and the team had no association with the research participants. Using G*Power 3.1, a sample size of N = 120–125 was determined. This calculation assumed a medium effect size (0.5), a power of 0.80, and an alpha of 0.05 and accounted for a 10% attrition rate. The parameters were based on the mean differences in post-traumatic growth reported in a prior study by Yilmaz et al. (2018) [28].

### 2.3. Ethical Considerations

Ethical approval was obtained from the Institutional Ethical Review Board (IRB# 2021-1156), and the study protocol was registered on ClinicalTrials.gov (NCT06674876) on 15 November 2021. This study was funded by the American Psychiatric Nurses Association, USA, and the University of East London, UK. Surveys were developed using Qualtrics software [29]. The pre-surveys at baseline included a participant consent form along with a detailed study information letter. The study information letter provided clear details about this study’s purpose, participant involvement and their rights, benefits/harms, intervention, random allocation, group assignments, data collection, duration, timelines, and the contact information of the university IRB and PIs for any questions or concerns. The letter included a helpline for suicide prevention and mental health crisis counselors for participants who might experience mental discomfort, anxiety, or stress during this study. Participants received an honorarium of USD 10 upon the completion of each survey and an additional USD 25 upon the completion of eight blog entries (two blog entries/week) for four weeks. The honorarium was sent to the participants through emails by a designated university representative.

### 2.4. Intervention

The intervention consisted of a 4-week blog writing program. Participants in the intervention group were assigned to write two blog entries/week for a duration of 4 weeks and complete surveys designed on Qualtrics software (https://www.qualtrics.com/) at 4 weeks and 6 weeks. A structured blog writing template was developed on Qualtrics software. Separate weekly Qualtrics links for writing blog entries were sent to participants in the intervention group. Participants were told to write two blog entries each week following the given template at their own convenient time, ideally at different times of the week. Participants were told that they could create blog entries in the form of words, stories, short poems, or artwork. Follow-up reminders were sent each week to complete these blog entries by the PI and the assigned research assistant who only had access to the participant emails.

The weekly blog template included short questions and prompts to generate self-reflections, ideas, and thoughts guided by the SOPHIE framework. **Week 1** focused on self-exploratory and ontological inquiry questions that can assist nurses in enabling congruency, transformation, and leadership in trans-cultural nursing practices. For example, in week 1, participants were encouraged to reflect on and write about their personal and professional identity constructs that influence their values and beliefs by considering questions such as the following: Who am I as a person? Who am I as a palliative care nurse? **Week 2** focused on the phenomenological and humanistic aspects of the SOPHIE framework. Participants were asked to explore their self-perceptions and subjective experiences of loss, pain, and suffering in their professional work setting. The phenomenological aspect was focused on the following question: How am I (engaging in this situation)? This component aimed to explore areas affecting the participants’ relational well-being and practice approach with service users, carers, and colleagues. The humanistic aspect considered the following question: What can I offer to others? Participants were asked to explore whether there were any issues with professional authenticity such as fear or doubt that may affect their self-determination, passion, and motivation to care and connect with others. **Week 3** asked about the ideological and existential aspects encouraged self-reflection on the following: How do I belong with others? It also asked about participants’ strengths, fears, and limitations with respect to connecting with and accepting others as they are. How do they relate to others who may come from a different religious, social, cultural, or linguistic background? For the existential aspect, the following question was considered: Why do I exist? The aim was to encourage participants to find the truth, meaning, and professional authenticity that influence their caregiving approaches as palliative care nurses. Week 4 focused on SOPHIE as Practice Wisdom—Participants were asked to draw or summarize their reflective account based on previous three-week log entries. The aim was to develop reflexivity among participants to navigate their identity construct around their personal and professional selves, explore their personal truth, and acknowledge the nature of trauma, fear, or vulnerability they may have been experiencing, as PC nurses. See Figure 1. 

### 2.5. Self-Reported Survey Measures

Following the study aims, self-reported survey measures such as the Subjective Well-Being Inventory (SUBI), Brief Resiliency Scale (BRS), and Post-traumatic Growth Inventory (PTGI) scales were used. All these scales have high reliability scores and have been used in different population groups. The SUBI scale is a five-item Likert scale (1 = strongly disagree to 7 = strongly agree) [30]. The BRS is a six-item Likert scale (1 = strongly disagree to 5 = strongly agree) [23,31,32]. Items number two, four, and six on the BRS were reverse-coded. PTGI is a 21-item Likert-type scale divided into 5 subscales: Relating to others (7 items), New Possibilities (5 items), Personal Strength (4 items), Spiritual Change (2 items), and Appreciation of Life (3 items). Each item was rated on a 6-point Likert scale, where 0 = I did not experience this change as a result of my crisis; 5 = I experienced this change to a very great degree as a result of my crisis [24,33]. Demographic variables in the survey included age, gender, ethnicity, income, educational qualification, designation at work, clinical placement, years of experience, and PC training.

### 2.6. Procedure and Data Collection

**Figure 2:**
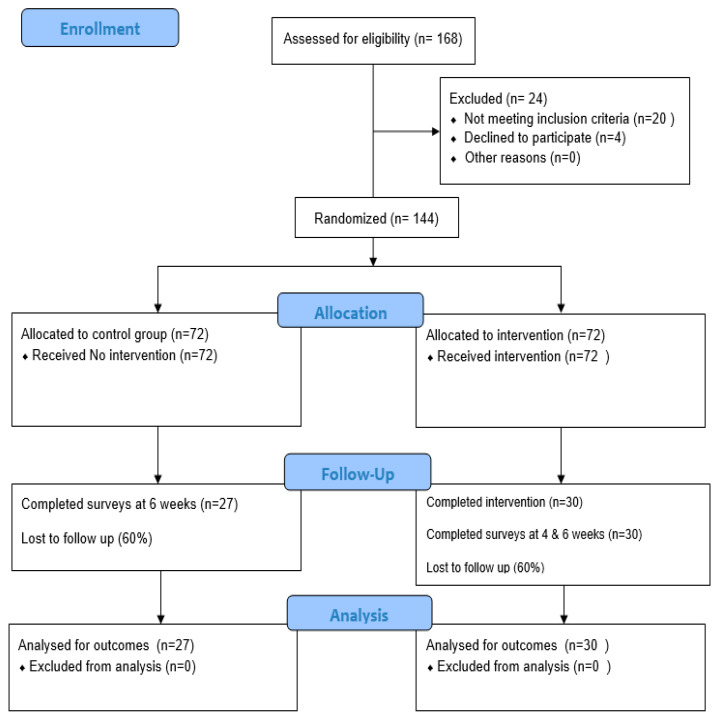
CONSORT 2025 flow diagram.

The following quality control measures were taken to ensure the accuracy and consistency of the data and intervention. A standard study protocol was developed. The research team consisted of expert researchers who had prior experience with intervention studies and had trained one undergraduate and one graduate student, ensuring a high level of competency in executing this study. A pilot test of the survey instruments was conducted with five participants to identify and rectify any potential issues before the full study commenced. Using member listservs, email invitations were sent to the eligible participants to enroll in this study and complete the anonymous survey at baseline. Participants were asked to create a unique ID using the first two letters of their parents’ names followed by two digits of their birthdate to track participants’ responses. A total of N = 144 participants completed the baseline surveys. Upon baseline survey completion, participants were randomly assigned to control (N = 72) and intervention groups (N = 72) using computer-generated randomization tools (see Figure 2). Participants in the intervention group were asked to write 2 blog entries/week for a duration of four weeks. All the surveys and blog entries were anonymous and contained the self-created IDs only provided by the participants at baseline. Separate weekly Qualtrics links and follow-up reminders for blog writing were sent through participant emails in the intervention group. Only the PI and a research assistant responsible for data collection had access to participant emails in both the groups. Participants in the control group completed the surveys at baseline and at 6 weeks, whereas participants in the intervention group completed the surveys at baseline and at 4 weeks and 6 weeks post-intervention. The post-surveys sent to the intervention group in week 4 and week 6 included four additional descriptive questions for any feedback or suggestions about the intervention used in this study. All the data was kept in an encrypted institutional Box folder, and only the research team had access to the data. As stated earlier, weekly reminders were sent to participants in both the groups for study completion. Regular biweekly research team meetings were conducted to plan and evaluate the study procedures and to review the completed surveys and blog entries for assessing and verifying data completeness and accuracy. A comprehensive audit trail was maintained throughout this study to ensure the rigor, transparency, and credibility of the research.

A flow diagram of the progress made through the phases of a randomized trial of two groups (that is, enrolment, intervention allocation, follow-up, and data analysis) is shown above.

## 3. Data Analysis

Statistical analyses were conducted using IBM SPSS Statistics for Windows, Version 27.0 [34]. Descriptive statistics, including means, standard deviations, and percentages, were used to summarize demographic variables. Missing data was handled using listwise deletion, which excludes cases with missing values from the analysis. This approach was chosen due to the low rate of missingness and to maintain consistency across statistical tests [35]. For outcome variables, two types of *t*-tests were employed: an independent samples t-test to compare mean differences in the three self-reported measures between the control and intervention groups before and after the intervention and a paired samples t-test to assess changes within the intervention group across the three time points. Additionally, an Analysis of Covariance (ANCOVA) was used to examine the effects of multiple independent variables and repeated measures within this study. Descriptive survey responses and blogs were analyzed using content analysis [35,36]. Narrative data obtained from the blogs was coded, and categories were formulated. Biweekly meetings and discussions were held among the research team members to come to a consensus about the themes generated from the blog narratives.

## 4. Results

A total sample of N = 144 participants was considered in this study. The control group included N = 72 participants, and the intervention group included N = 72 participants. Only N = 57 participants completed this study, with an attrition rate of 60%. Within the control group, N = 27 participants completed the surveys at baseline and at 6 weeks, whereas within the intervention group, only N = 30 participants completed the surveys at baseline, 4 weeks, and 6 weeks and wrote eight blog entries, i.e., two/week for a period of 4 weeks.

### 4.1. Demographic Variables

Most participants were females (93%), White (89.5%), and married (93%). About 70% were between the ages of 29 and 39 years, had a bachelor’s degree (84%), were full-time employees (96.5%), and had undergone PC training (93%). Table 1 presents the demographic characteristics of all the participants. Table 2 and Table 3 provide the results for mean differences across the three study outcomes, i.e., subjective well-being, resilience, and post-traumatic growth.

**Figure 3:**
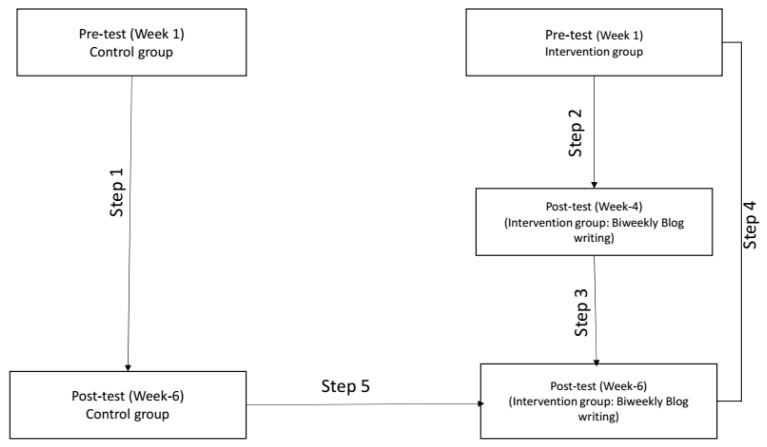
Stepwise flowchart for outcome analysis among different study groups.

The mean differences in the study outcome measures were tested at a significance level of *p* < 0.05 in five steps: (i) pre-test and post-test phases within the control group from week 1 to week 6; (ii) pre-test and post-test phases within the intervention group from week 1 to week 4; (iii) post-test phase within the intervention group from week 4 to week 6; (iv) comparison of pre-test and post-test within the intervention group from week 1 to week 6; and (v) comparison of post-test mean differences between the intervention and control groups at week 6. Refer to Figure 3.

**Subjective Well-Being (SUBI)**: The findings indicate significant mean differences in the subjective well-being (SUBI) scores (MD = 2.43, *p* < 0.05) at week 6 post-intervention in the intervention group compared to the control group. Within the intervention group, SUBI was found to be significant across all study points (*p* < 0.05). Refer to Table 2.

**Resilience (BRS)**: A significant difference was found in the resilience scores at baseline and at 4 weeks in the intervention group (MD= 0.244, *p* < 0.05) as compared to the control group. No differences were found at week 6 in either the intervention or control group.

**Post-traumatic Growth Inventory (PTGI)**: There was no significant difference in the PTGI mean scores at any study point in both the groups.

Table 4 presents the results of the ANCOVA, which examined differences between the two groups while controlling for demographic covariates, including age, years of experience, and prior PC training. The results indicate that years of experience in PC (F (1, 80) = 6.34; *p* = 0.01) and the time of measurement (F (1, 80) = 7.03; *p* = 0.01) were found to be significant factors affecting the SUBI outcomes in both the groups. Resilience scores, however, were not influenced by the covariates included in the analysis and were affected only by the time of measurement (i.e., at baseline, week 4, or week 6) (F (1, 81) = 4.39; *p* = 0.04). The R-squared values suggest a moderate proportion of variance explained in post-traumatic growth and subjective well-being, while a smaller portion of the variance in resilience was accounted for.

Acceptability: Post-surveys included four open-ended questions that asked participants for their feedback and suggestions to make further improvements to this study. All participants (100%) said that the intervention was beneficial. It helped them to reflect on their personal and professional experiences, vulnerabilities, feelings, and emotions. No adverse events were reported by the participants. This demonstrates the acceptability of intervention.

### 4.2. A Qualitative Analysis of the Blog Narratives: Given Below Are the Themes Generated from the Weekly Blog Narratives Completed by the Intervention Group

#### 4.2.1. Self-Awareness and Reflection

Nearly all participants said that blog writing was helpful and generated self-awareness, personal reflections, and healing. Participants used phrases like ‘it was helpful for their mental journey’; it helped them to ‘deepen understanding of own self’; it helped them in terms of a ‘reflection on self-growth’; and it helped them to ‘record footprints’, daily emotions, and mood. It was interesting to note that the word ‘healing’ was used more than 20 times in participants’ narratives. Blog writing helped them to reflect on their moral distress, generated insight, and promoted self-growth. Participants deemed the intervention a meaningful, therapeutic, helpful, and supportive activity. Below are some quotes from the narratives:

*I believe some healing took place and has continued in my understanding of past experiences and my work.*

*It allows me to think more calmly and deal with the trauma I have encountered, giving me a better meaning in life. it helped me heal the trauma am experiencing. Help me in stress relief, help me to better stabilize my emotions. Helped me reflect on my past which had a great effect on me. It helped make my time useful and helped me come out of this mess. Feel calm.*

*Writing about traumatic events can help ease the emotional stress of negative experiences… I feel good when I reflect on the trauma I’ve been through and connect and heal through SOPHIE.*

#### 4.2.2. Positive Attitude Towards Life

Participants expressed having a ‘calmer self’ and feeling ‘happy’. Writing blog entries helped them in generating self-awareness, such as by telling themselves ‘not to ignore the trauma’ and trying ‘better ways to heal wound’. These exercises allowed them to rethink their values, perspectives, and worldviews and motivated them to develop a positive attitude towards life. Below are some quotes from the participants:

*SOPHIE helped me to take responsibility for my thoughts, find meaning in trauma, stay open to myself, and develop new worldviews.*

*Helped me do a life review. I could reevaluate where some change is needed for better balance and happiness.*

#### 4.2.3. Find Meanings and Connections Within Self

Blog writing helped the participants to identify their fears and anxieties and enabled them to reconnect with themselves and find meanings in their actions. A few quotes are presented below:

*Using SOPHIE made me feel vulnerable sometimes. Vulnerability is also a sign of courage. When we embrace who we are and how we feel, we become more resilient and braver.*

*Writing blogs made me strong and helped me to reevaluate my values and beliefs. it was a kind of therapeutic journal which I haven’t done in a while.*

## 5. Discussion

Our study evaluated the feasibility of a structured blog writing intervention for improving the subjective well-being, resilience, and post-traumatic growth of PC nurses during the COVID-19 pandemic. Our findings showed a significant improvement in participants’ subjective well-being and resilience post-intervention. The use of a Qualtrics platform to deliver the expressive writing intervention was successful and well received by the participants. Overall, participants reported that the blog writing intervention was helpful and enabled them to express their thoughts and feelings safely and conveniently. Similar results have been reported in other studies also [37,38,39]. Blog writing interventions carry multiple practical merits as they are non-invasive and inexpensive and present minimal risks for participants. They offer increased privacy and anonymity and encourage more personal space for emotional expression and self-reflection [22]. Our findings suggest that blog or self-reflexive writing interventions guided by the NPT and SOPHIE frameworks can be feasibly and effectively delivered using Qualtrics, an online virtual platform.

Resilience plays a substantial role in promoting subjective well-being and post-traumatic growth [40]. Our findings indicate a significant improvement in participants’ resilience immediately at week 4 post-intervention. Another feasibility study during COVID-19 also showed similar results [41]. The authors used an online expressive writing intervention for six weeks among community adults and found a significant improvement in their resilience and post-traumatic growth [41]. This study did not show any significant improvement in post-traumatic growth, possibly because the intervention was implemented for a short period of time, i.e., four weeks only. Evidence suggests that post-traumatic growth is a gradual process, and longer intervention periods may be necessary to observe significant changes among participants. Previous studies among nurses suggest that higher resilience scores correlate with greater PTGI scores, indicating that nurses with higher resilience demonstrate positive psychological growth and better coping and well-being following a traumatic experience or adverse situation [28,42,43,44,45]. While the relationship between resilience and post-traumatic growth has been established in the prior literature, most of these studies have used a psychoeducational approach or multimodal intervention strategies over a longer period, i.e., more than four weeks. This also suggests that post-traumatic growth or positive psychological transformation following trauma or adversity is an ongoing process and not a static outcome [24]. Future studies are therefore recommended.

The qualitative data gathered from the blog narratives revealed that most participants found writing blog entries helpful and viewed it as a process of self-expression, self-discovery, and disclosure [26]. Writing blog entries allowed individuals to reflect on their strengths and weaknesses, process their self-regulation abilities, and improve their self-confidence. Through this self-reflection, they were able to comprehend and find meaning in their roles, which provided a sense of personal and professional fulfillment [26,46]. Blog writing kept them focused and engaged, allowing them to form deep and lasting connections with themselves and others. Furthermore, the intervention empowered them to make sense of their inner and outer worlds while encouraging a positive outlook on life and the achievement of personal transformation. These qualitative findings are, however, subjective in nature and need further in-depth exploration. Future research is recommended to evaluate the effectiveness of self-reflexive blog writing interventions in a larger population across varied practice settings.

### Study Limitations

There were several study limitations. This study included participants from several PC and healthcare institutions within the US regardless of their specific geographical location. This recruitment strategy may have inadvertently allowed for the possibility that some participants may have also been involved in other psychoeducational or supportive programs, potentially affecting the study results. The self-reported outcome measure scales were low-burden given the low number of items included; however the blog writing frequency and the length of this study may be limitations given the busy schedule of practicing nurses esp. during the pandemic. The post-surveys among the control group were only conducted at baseline and 6 weeks, highlighting the need for uniform data collection schedules in future research. There is a likelihood of retrospective recall bias in the participants’ blog responses. There was a high attrition rate of 60% in this study despite the weekly follow-up reminders. This may be due to the timing of this study as it was conducted during the COVID-19 pandemic period. Future studies need to consider timing of the study, uniform data collection points, and strategies to minimize attrition rates.

## 6. Conclusions

PC nurses are at risk of poor mental health and well-being. Self-reflexive blog writing is a convenient, flexible, and low-cost intervention that can improve their subjective well-being, resilience, and post-traumatic growth. Nurse managers and organizational leaders should adopt these supportive measures to improve nurses’ retention, satisfaction, and overall quality of life.

## Figures and Tables

**Table 1:** Participants’ demographic characteristics at baseline.

Baseline Characteristic	Total (N = 57)		Control Group (N = 27)		Intervention Group (N = 30)	
	N	%	N	%	N	%
**Gender**						
Female	53	93	26	96.3	28	93.3
Male	4	7	1	3.7	2	6.7
**Marital status**						
Single	2	3.5	1	3.7	5	16.7
Married/partnered	53	93	26	96.3	24	80
Divorced/widowed	1	1.8	-		1	3.3
**Age group**						
18–28	3	5.3	1	3.7	2	6.7
29–39	40	70.2	18	66.7	21	70
40–50	12	21.1	8	29.6	6	20
≥62	1	1.8	-	-	1	3.3
**Nursing Education level**						
Associate degree	9	15.8	9	33.3	11	36.7
Bachelor’s degree	48	84.2	18	66.7	19	63.3
**Employment**						
Employed full-time	55	96.5	22	81.5	30	100
Employed part-time	2	3.5	5	18.5	-	-
**Race**						
African American	6	10.5	6	22.2	2	6.7
White	51	89.5	21	77.8	28	93.3
**Ethnicity**	6	12				
Hispanic	3	5.3	3	11.1	-	-
Non-Hispanic	54	94.7	24	88.9	30	100
**Household Income**						
USD 35,000–USD 49,000	28	49.1	10	37	2	6.7
USD 50,000–USD 74,999	20	35.1	16	59.3	2	6.7
USD 75,000–USD 99,900	5	8.8	1	3.7	23	76.7
Over USD 100,000	4	7	-	-	3	10
**Palliative Care Training**	53	93	27	100	29	96.7

**Table 2:** Mean difference (MD) in outcome measures (within subjects—N = 57).

**Mean Differences (MDs) Within Control Group (N = 27)**													
**Steps**	**PTGI**				**SUBI**				**BRS**				
	** MD **	***t***	***df***	***p***	** MD **	***t***	***df***	***p***	**Mean**	***t***	***df***	***p***	**Cohen’s d**
Step1: Pre/Post-6 weeks	−3.42	−1.0	25	0.16	0.62	−0.34	26	0.37	−0.02	−0.13	26	0.89	−0.18/0.00370
**Mean Differences (MDs) Within Intervention group (N = 30)**													
	**PTGI**				**SUBI**				**BRS**				
	**MD**	***t***	***df***	***p***	**MD**	***t***	***df***	***p***	**MD**	***t***	***df***	***p***	**Cohen’s d**
Step2: Pre/Post-4 weeks	−1.6	−0.33	29	0.37	−0.07	2.58	28	0.001 *	0.24	2.45	29	0.02 *	−0.060/0.0037
Step 3: Post-4 weeks–Post-6 weeks	2.86	1.23	29	0.11	1.10	3.97	28	0.001 *	−0.02	−0.13	26	0.89	0.225/0.0496
Step 4: Pre/Post-week 6	1.27	0.29	29	0.38	1.73	6.72	28	0.008 *	0.18	−0.81	29	0.17	0.053/0.0029

* *p* < 0.05.

**Table 3:** Mean differences between groups (N = 57).

Step 5: Mean Differences (MDs) Between Groups	MD (SD)	*t* (df)	*p*	Cohen’s d
PTGI—Intervention	72.11(9.79)	0.751 (52)	0.456	0.205/0.011
Control	69.92 (11.55)			
BRS—Intervention	3.03 (0.314)	−0.093 (55)	0.927	−0.025/0.0002
Control	3.04 (0.48)			
SUBI—Intervention	2.43 (1.16)	−4.83 (49)	<0.001 *	1.279/0.323
Control	3.67 (0.73)			

* *p* < 0.05 (N = 27 for control group; N = 30 for intervention group). Note: Results were considered with equal variance assumed.

**Table 4:** Mean differences between groups while controlling demographic covariates (N = 57).

Outcomes	Sum of Squares	df	Mean Square	*F*	*p*	η^2^_p (Effect Size)_
**PTGI**						
Age	719.88	1	719.88	3.45	0.06	0.050
Income	542.99	1	542.99	2.60	0.11	0.039
Clinical area	499.72	1	499.72	2.39	0.12	0.036
Years as RN	91.02	1	91.02	0.43	0.51	0.007
Designation at work	9.70	1	9.70	0.04	0.83	0.001
Years in palliative care	164.64	1	164.64	0.78	0.37	0.012
Time of measurement	530.80	1	530.80	2.54	0.11	0.038
Total	19,501.44	77				
R-squared = 0.305 (adjusted R-squared = 0.176)						
**Subjective Well-Being**						
Age	23.75	1	23.75	1.73	0.19	0.025
Income	12.59	1	12.59	0.92	0.34	0.013
Clinical area	19.75	1	19.75	1.44	0.23	0.021
Years as RN	8.76	1	8.76	0.64	0.42	0.009
Designation at work	31.50	1	31.50	2.30	0.13	0.033
Years in palliative care	86.67	1	86.67	6.34	0.01 *	0.085
Time of measurement	96.18	1	96.18	7.03	0.01 *	0.094
Total	1328.000	80				
R-squared = 0.300 (adjusted R-squared = 0.177)						
**Resilience**						
Age	0.38	1	0.38	1.41	0.23	0.020
Income	0.12	1	0.12	0.47	0.49	0.007
Clinical area	0.01	1	0.01	0.04	0.82	0.001
Years as RN	0.18	1	0.18	0.70	0.40	0.010
Designation at work	0.42	1	0.42	1.57	0.21	0.022
Years in palliative care	0.000	1	0.000	0.000	0.98	0.000
Time of measurement	1.17	1	1.17	4.39	0.04 *	0.060
Total	20.985	81				
R-squared = 0.117 (adjusted R-squared = −0.037)						

* *p* < 0.05. Note: Interaction effects were not included as none were significant.

## Data Availability

The original contributions presented in this study are included in the article. Further inquiries can be directed to the corresponding author.

## Abbreviations

ANCOVA	Analysis of Variance
BRS	Brief Resiliency Scale
IRB	Institutional Ethical Review Board
NPT	Nurses’ Psychological Trauma
PC	Palliative care
PTSD	Post-Traumatic Stress Disorder
PTGI	Post-Traumatic Growth Inventory
RSL	Resilience
SOPHIE	Self-exploration on Ontological, Phenomenological, Humanistic, Ideological, and Existential
SPSS	Statistical Package for the Social Sciences
SUBI	Subjective Well-Being Inventory
US	United States
